# One–Step Synthesis of Three–Dimensional Na_3_V_2_(PO_4_)_3_/Carbon Frameworks as Promising Sodium–Ion Battery Cathode

**DOI:** 10.3390/nano13030446

**Published:** 2023-01-21

**Authors:** Lijiang Zhao, Xinghua Liu, Jinsong Li, Xungang Diao, Junying Zhang

**Affiliations:** 1 School of Physics, Beihang University, Beijing 100191, China; 2 School of Energy and Power Engineering, Beihang University, Beijing 100191, China

**Keywords:** Na_3_V_2_(PO_4_)_3_, holey-carbon frameworks, cathode, sodium-ion battery

## Abstract

Sodium–ion batteries (SIBs) are essential for large–scale energy storage attributed to the high abundance of sodium. Polyanion Na_3_V_2_(PO_4_)_3_ (NVP) is a dominant cathode candidate for SIBs because of its high-voltage and sodium superionic conductor (NASICON) framework. However, the electrochemical performance of NVP is hindered by the inherently poor electronic conductivity, especially for extreme fast charging and long-duration cycling. Herein, we develop a facile one-step in-situ polycondensation method to synthesize the three-dimensional (3D) Na_3_V_2_(PO_4_)_3_/holey-carbon frameworks (NVP@C) by using melamine as carbon source. In this architecture, NVP crystals intergrown with the 3D holey-carbon frameworks provide rapid transport pathways for ion/electron transmission to increase the ultrahigh rate ability and cycle capability. Consequently, the NVP@C cathode possesses a high reversible capacity of 113.9 mAh g^−1^ at 100 mA g^−1^ and delivers an outstanding high–rate capability of 75.3 mAh g^−1^ at 6000 mA g^−1^. Moreover, it shows that the NVP@C cathode is able to display a volumetric energy density of 54 Wh L^−1^ at 6000 mA g^−1^ (31 Wh L^−1^ for NVP bulk), as well as excellent cycling performance of 65.4 mAh g^−1^ after 1000 cycles at 2000 mA g^−1^. Furthermore, the NVP@C exhibits remarkable reversible capabilities of 81.9 mAh g^−1^ at a current density of 100 mA g^−1^ and 60.2 mAh g^−1^ at 1000 mA g^−1^ even at a low temperature of −15 °C. The structure of porous carbon frameworks combined with single crystal materials by in-situ polycondensation offers general guidelines for the design of sodium, lithium and potassium energy storage materials.

## 1. Introduction

Lithium–ion batteries (LIBs) have dominated the market in portable technology for decades due to their high energy density and power density [1,2,3]. However, further application is hindered by the scarcity and uneven distribution of lithium resources. Therefore, the search for low–cost, high performance alternatives has become a formidable challenge. Among the candidate batteries, sodium ion batteries are essential for large–scale electrochemical energy storage due to the high abundance of sodium, wide working temperature range and high safety [4,5]. It is significant to develop electrode materials for sodium batteries with fast sodium ion (Na^+^) migration characteristics and high structure stability. To date, numerous cathodes for SIBs such as NASICON-structured compounds [6], phosphates [7], Prussian blue analogues [8], layered transition and metal oxides [9] have been studied in depth. Among them, Na_3_V_2_(PO_4_)_3_ (NVP) shows unique advantages as one of the typical NASICON-structured materials [10,11,12,13]. It consists of VO_6_ octahedra and PO_4_ tetrahedra, which share all their corners to form a 3D open framework structure with numbers of alkali-ion interstitial sites, possessing high voltage platform, high safety, long cycle stability and good thermal stability [14]. Nevertheless, the valence electrons of transition metals within the lattices are isolated by the electronically insulating phosphate groups, which causes poor intrinsic electronic conductivity [15]. To solve this issue, synthesizing nanostructured NVP, constructing a porous hollow structure, and coating conductive materials are the most common strategies [16,17,18]. Xu et al. [19] fabricated porous NVP/reduced graphene oxide hollow spheres (NVP/rGO HSs) by the spray drying method and achieved a high capacity of 116 mA h g^−1^ at 1C and stable cycle stability of 73.1 mAh g^−1^ after 1000 cycles at 10 C. Lin et al. [20] reported Na_3_V_2_(PO_4_)_3_ of hollow porous microspheres and carbon-based framework by a microalgae-based biochemistry-directed bottom-up method that revealed a superior capability of 112 mAh g^−1^ at 0.2 C. Cong et al. [21] wrapped Na_3_V_2_(PO_4_)_3_ by ZIF–67–derived carbon using the sol-gel and solid-phase method to achieve improved electrical conductivity and electrochemical performance. In addition to the carbon layer that provides a favourable electronic transport pathway, the high electrochemical performance of the above materials benefits from the large-pore and hollow spheres framework with high ionic diffusivity. Nevertheless, these large-pore open frameworks cannot store lots of alkali metal atoms per volume, and are therefore not the best choice for high volumetric energy density cathodes with fast charging [22]. Micron-sized crystals can improve the volume energy density, while the delayed Na^+^ diffusion coefficient and electron conductivity will lead to the decay of specific capacity. Therefore, it is necessary to enhance the electron/ion transmission of NVP and reduce the porosity of electrode materials through reasonable structural design.

Herein, we report a 3D Na_3_V_2_ (PO_4_)_3_/holey-carbon framework as a promising cathode material for SIBs synthesized using a one-step synthesis method by in-situ polycondensation melamine. The holey-carbon layer connected with NVP crystals forms a network for the conductive scaffold, and the porous structure enables rapid mass transport, thereby facilitating the penetration and fast diffusion of sodium ions, as well as satisfying the electrode compaction density. Therefore, NVP@C displays impressive electrochemical performance, including high reversible capacity (113.9 mAh g^−1^ at 100 mA g^−1^, 0.9 C), excellent rate capacity (75.3 mAh g^−1^ at 6000 mA g^−1^, 80 C), improved volumetric energy density (54 Wh L^−1^ for NVP@C, 31 Wh L^−1^ for NVP bulk, 6000 mA g^−1^), superior cycling stability (65.4 mAh g^−1^ after 1000 cycles at 2000 mA g^−1^, 20 C), and outstanding rate performance at low temperature (81.9 mAh g^−1^ at 100 mA g^−1^ and 60.2 mAh g^−1^ at 1000 mA g^−1^, −15 °C). The design to form a composite by coupling 3D holey-carbon frameworks and nanocrystal by the one-step method may open up a new direction into the construction of fast-charging energy storage materials.

## 2. Materials and Methods

### 2.1. Synthesis of NVP@C and NVP Bulk

All chemicals for synthesizing NVP@C and NVP bulk in laboratory were purchased from Macklin (Macklin Inc., Shanghai, China). According to stoichiometric amount, the raw materials of NH_4_VO_3_ (1.872 g, 99.95% metals basis), NH_4_H_2_PO_4_ (2.761 g, AR, 99%), Na_2_CO_3_ (1.272 g, AR, 99%) and Melamine (1.4 g, 99%) were mixed in deionized water and a 70 mL solution was prepared. Then the mixture was freeze dried and annealed in flowing Ar at 700 °C for 10 h with a heating rate of 1.5 °C min^−1^ to obtain the NVP@C materials. The synthetic method of NVP bulk was similar to NVP@C except that melamine was not included.

### 2.2. Characterization of Materials

A Bruker D8 Advance X–ray diffractometer (Cu Kα radiation λ = 1.5418 Å, Bruker Inc., Karlsruhe, Germany) was used to collect the powder X–ray diffraction (XRD) patterns over the 2 Theta range of 10–50°. The morphology and size of the specimens were characterized by field emission scanning electron microscope (FESEM, Gemini SEM 500, Carl Zeiss AG Inc., Oberkochen, Germany) coupled with energy dispersive X–ray (EDS) analysis. The detailed nanostructures of the products were collected by transmission electron microscope (TEM, JEM–2100F, JEOL Inc., Shoshima, Tokyo, Japan) performing at 200 KV. NETZSCH STA 449 F5/F3 Jupiter (NETZSCH Inc., Bavaria, Germany) was employed to record the thermogravimetric (TG) curves of the samples with a heating rate of 10 °C min^−1^ from 25 °C to 1000 °C in air. Renishaw in Via (Renishaw Inc., Gloucestershire, UK) was employed for the Raman spectroscopy characterization with a 532 nm laser. X-ray photoelectron spectroscopic (XPS) data were obtained by Thermo ESCALAB 250Xi (Thermo Fisher Scientific Inc., Waltham, MA, USA).

### 2.3. Electrochemical Measurements

The cathode was a mixture dispersed by N–methyl–2–pyrrolidone (NMP) including electroactive materials (NVP@C and NVP bulk), carbon conductor (Super P) and polyvinylidene fluoride (PVDF) with a weight ratio of 80:10:10. The paste was spread on the Al foil, then dried at 70 °C for 4 h in an oven and finally baked at 110 °C overnight in a vacuum oven. In this work, the mass loading of electroactive materials was around 1.0–2.0 mg cm^−2^. The electrochemical performance of half-cell was investigated using coin-type cells (CR 2032), with sodium metal as reference and counter electrodes. The separator is a glass microfiber filter (CAT No. 1820–047, Whatman Inc., Maidstone, UK). The diameter is 12 mm for cathode electrodes including NVP@C and NVP bulk. As the anode electrode, the diameter of Na metal is also 12 mm. The electrolyte was 1.0 M NaClO_4_ in a 1:1:1 (Vol%) mixture of dimethyl (DMC), ethylene carbonate (EC) and ethyl methyl carbonate (EMC). All batteries were assembled in an Ar filled glove box with oxygen and water contents below 0.01 and 0.01 ppm, respectively.

### 2.4. Electrochemical Characterization

All charge/discharge cycles were tested with the voltage range of 2.0~4.0 V for the cathode (NVP@C and NVP bulk)/Na half cells using LAND 2001 A Cell test system (LANHE Inc., Wuhan, China). And electrochemical analyzer (CHI 660, chinstruments Inc., Shanghai, China) was used to conduct cyclic voltammetry (CV), electrochemical impedance spectroscopy (EIS) and floating test. The EIS tests at 2.0 V were carried out from 0.01 to 100 K Hz with a perturbation of 5 mV applied over the frequency range.

## 3. Results and Discussion

### 3.1. Synthesis and Structural Characterization

**Figure 1:**
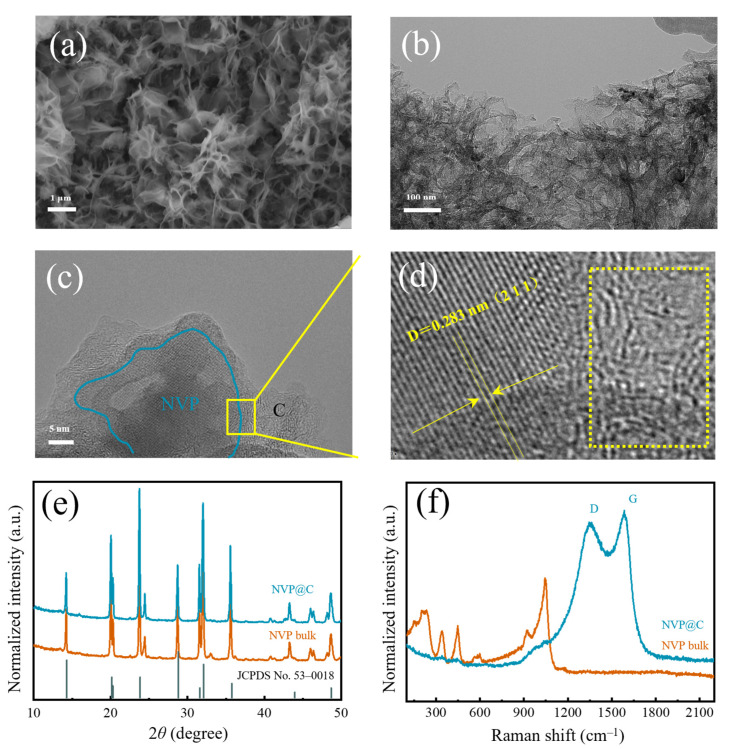
(**a**) SEM image, (**b**) TEM image, (**c**) HRTEM image and (**d**) magnification region (amorphous carbon is marked in the dotted box) of NVP@C; (**e**) XRD patterns and (**f**) Raman spectra of NVP@C and NVP bulk.

In the experiments, melamine is employed as sacrificial template via a facile in-situ construction method to obtain a 3D hierarchical holey Na_3_V_2_(PO_4_)_2_ framework (NVP@C). The scanning electron microscopy (SEM) images of NVP@C illustrate a 3D interconnected, coral-like morphology (Figure 1a and Figure S1) after annealing in Ar. The structure of NVP@C can be obviously distinguished in the transmission electron microscope (TEM) image (Figure 1b). Obviously, after uniform mixing and annealing in Ar, melamine is decomposed and leaves a 3D hierarchical holey-carbon framework, which is composed of cross-linked curly nanosheets with a size of 3–5 μm, and nanoparticles. Typical high–resolution TEM (HRTEM) images of NVP@C in Figure 1c indicate that NVP nanocrystals (~50 nm) are dispersed in the 3D hierarchical holey-carbon framework homogeneously (Figure S2). The selected area electron diffraction (SAED, Figure S2d) pattern confirms the crystallinity of the NVP nanocrystals. The lattice spacing of 0.283 nm can be identified as (211) interplanar spacing of NVP, while the lattice fringe in the rightregion is amorphous carbon (Figure 1d). The intergrowth structure is favorable for fast electronic transmission and infiltration of the electrolyte. Figure 1e shows the X–ray diffraction (XRD) pattern of the prepared NVP@C and NVP bulk. The diffraction peaks of both NVP@C and NVP bulk can be well conformed to the Na_3_V_2_(PO_4_)_2_ phase (JCPDS No. 53–0018), which suggests that the 3D interconnected holey-carbon frameworks do not affect the phase purity of NVP. The crystallite size of NVP@C (~50 nm) and bulk NVP (~50 nm) are calculated by Scherrer Formula, implying the carbon framework could reduce grain cluster and disperse the crystals well. The existence of the carbon element can be confirmed by Raman spectrum (Figure 1f). Two newly emerged Raman peaks at 1354 cm^−1^ and 1587 cm^−1^ belong to disorder induced D–band and graphitic G–band, respectively. The intensity ratio of the two peaks (I_G_/I_D_) is about 1.2 [23,24], verifying the existence of a considerable amount of graphitized carbon, thus facilitating the electron transport.

**Figure 2:**
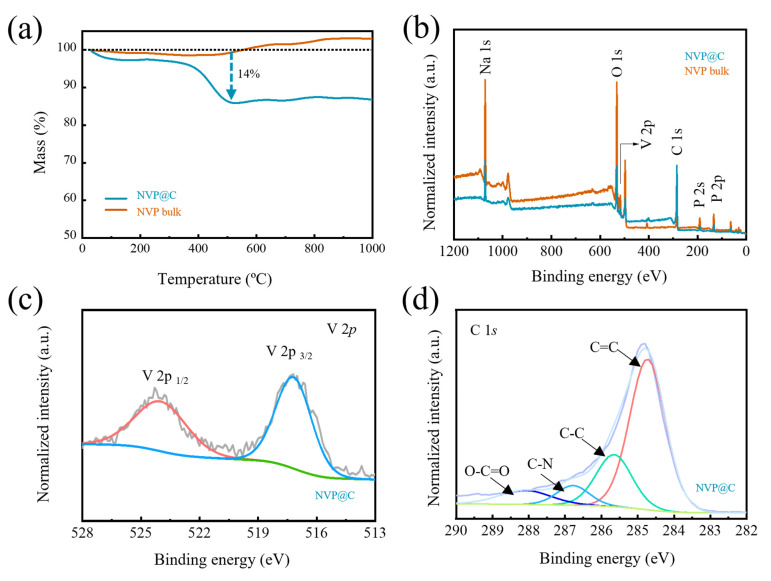
(**a**) TG curves of NVP@C and NVP bulk; (**b**) XPS full spectra, (**c**) V 2p spectra and (**d**) C 1s spectra of NVP@C and NVP bulk.

Thermogravimetric (TG) analysis curves determine that the carbon content in NVP@C equals 14% from the weight loss below 500 °C (Figure 2a). The existence of the holey-carbon coating layer can be furthermore confirmed by X–Ray photoelectron spectroscopy (XPS) tests. The characteristic peaks of Na, O, V, C and P can be clearly obtained from the full XPS spectra (Figure 2b). Two fitted peaks located at 516.6 and 523.6 eV are ascribed to V 2p_3/2_ and V 2p_1/2_ of V^3+^ ion (Figure 2c) [25]. The P 2p peak and O 1s are also obtained at 132.8 eV and 531.1 eV (Figure S3), confirming the existence of PO_4_^3−^ [26]. The high-resolution C 1s spectrum of NVP@C is shown in Figure 2d and Figure S3, and four peaks at 284.7, 285.6, 286.7, 288.1 eV belong to C=C, C–C, C–N, and O=C–O, respectively [27]. Remarkably, the characteristic peaks in NVP@C are blunt compared with NVP bulk, except for C 1s spectrum, for holey–carbon coating shields part of the signals of NVP. Therefore, the XPS results confirm that a carbon layer was coated on NVP.

### 3.2. Electrochemical Properties of NVP@C and NVP Bulk

**Figure 3:**
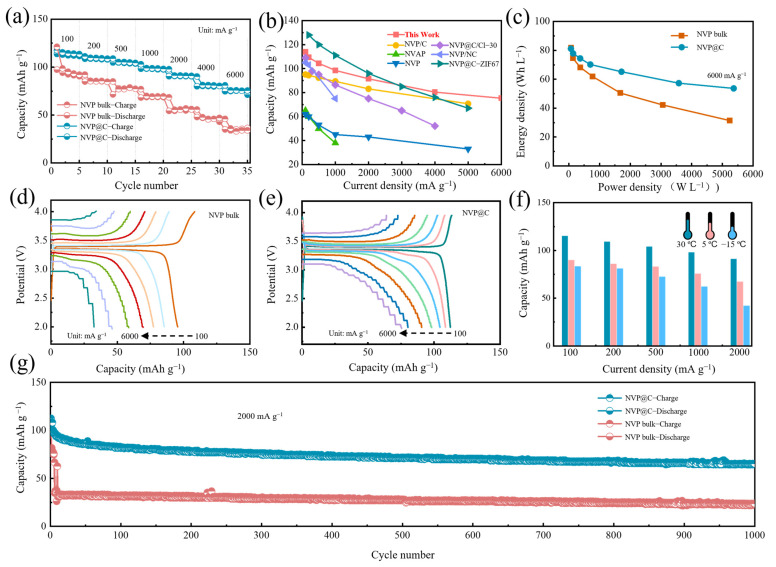
(**a**) Rate capability of NVP@C and NVP bulk; (**b**) comparison of the rate capability of NVP@C with other reported NVP-based materials such as cathode (NVP/C [26], NVAP [29], NVP [30], NVP@C/Cl–30 [31], NVP/NC [32] and NVP@C–ZIF67 [21]); (**c**) Ragone plot of NVP bulk and NVP@C half–cell. Charge-discharge profiles of (**d**) NVP bulk and (**e**) NVP@C from 100 mA g^−1^ to 6000 mA g^−1^; (**f**) rate capability of NVP@C at 30 °C, 5 °C and −15 °C; (**g**) cycle stability of NVP@C and NVP bulk at 2000 mA g^−1^.

The electrochemical performance was firstly monitored by CR2032 coin cells with Na foil as the counter/reference electrode. Figure 3a exhibits the rate performance of NVP@C and NVP bulk under varying current densities. Apparently, the NVP@C electrode reveals ultrahigh rate performance with high capacity of 113.9 mA h g^−1^ (0.9 C), 109.3 mAh g^−1^ (2 C), 104.4 mAh g^−1^ (5 C), 98.5 mAh g^−1^ (10 C), 91.5 mAh g^−1^ (20 C), 80.4 mAh g^−1^ (60 C) and 75.3 mAh g^−1^ (80 C) at 100 mA g^−1^, 200 mA g^−1^, 500 mA g^−1^, 1000 mA g^−1^, 2000 mA g^−1^, 4000 mA g^−1^ and 6000 mA g^−1^, respectively, which is superior to that of the NVP electrode. As shown in the capacity versus current density plot in Figure 3b, NVP@C expresses excellent performance among most NVP–based composite materials at various current densities, especially at large current density. The NVP@C exhibits an electrode compaction density of 0.75 g cm^−3^, which is slightly lower than NVP bulk (0.98 g cm^−3^) because of the increased porosity. However, the calculated volumetric energy density [28] (current density: 6000 mA g^−1^) for NVP@C is 54 Wh L^−1^, much higher than that of NVP bulk (31 Wh L^−1^) (Figure 3c). This result clearly confirms that this structure related volume and specific capacity should be considered for the design of high–performance cathode. The comparison of the representative charge/discharge curves at a series of rates for the NVP bulk (Figure 3d) and NVP@C (Figure 3e) clearly demonstrate the overwhelming preponderance of the NVP@C cathode at various current densities, especially at high rates. Concretely, compared to the charge and discharge plots of NVP@C, the voltage intervals of the NVP bulk are progressively aggravated, for which the incremental electrochemical polarization and charge transfer impedance are mainly responsible. Furthermore, the splendid rate abilities of the NVP@C can be demonstrated from the low temperature at 5 °C and −15 °C. The cathode exhibits remarkable high–rate capabilities of 81.9 mAh g^−1^ at 100 mA g^−1^ and 60.2 mAh g^−1^ at 1000 mA g^−1^ (−15 °C) (Figure 3f and Figure S4). The NVP@C demonstrates desirable long–term cycling behavior at 2000 mA g^−1^ (30 °C) (Figure 3g). Remarkably, a reversible discharge capacity of 65.4 mAh g^−1^ can still be reserved with high coulomb efficiency at nearly 100% after 1000 cycles, while the NVP bulk can only remain at 23.7 mAh g^−1^ under the same circumstances (Figure 3g), indicating the lower overpotential during charge and discharge process. The high quality integrated conductive network can enhance the electrochemical performance due to greatly improved intrinsic conductivity, which promotes fast electronic transmission, improves high–rate capabilities and facilitates cycle stability.

### 3.3. Electrochemical Mechanism

**Figure 4:**
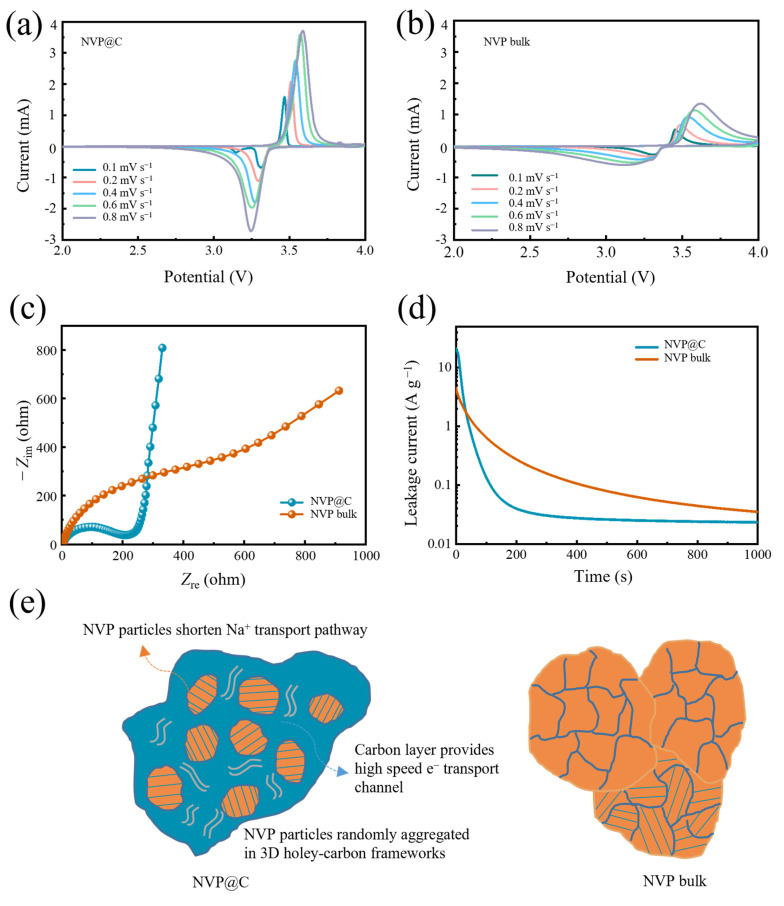
(**a**) NVP@C and (**b**) NVP bulk at rates from 0.1 to 0.8 mV s^−1^; (**c**) Nyquist plots of NVP@C and NVP bulk for the frequencies ranging from 0.01 to 100K Hz during discharge state; (**d**) leakage current of NVP@C and NVP bulk in floating tests; (**e**) schematic transport process of Na^+^ in NVP@C and NVP bulk.

To reveal the Na^+^ insertion/extraction kinetics of the NVP@C electrodes, we first gain cyclic voltammetry (CV) measurements ranging from 2.0 to 4.0 V. Figure 4a,b demonstrate the CV curves recorded at a series of scan rates from 0.1 to 0.8 mV s^−1^ for NVP@C and NVP bulk electrodes, respectively. The pair of typical redox peaks located at 3.4 V represent the transformation reaction between V^3+^ and V^4+^, corresponding to the two–phase transition reaction (i.e., Na_3_V_2_(PO_4_)_3_⇔NaV_2_(PO_4_)_3_) [19,29,33]. Furthermore, the higher peak current intensity also indicates the fast Na^+^ diffusion kinetics in NVP@C, which is due to the holey-carbon framework that enables high-speed ionic and electronic transportation. As the scan rates increase, the sharp characteristic redox peaks for NVP@C have been well reserved, suggesting an exceptional rate performance. The redox couple profiles of NVP@C are more compact, especially in comparison with those of the NVP bulk, implying enhanced redox kinetics during the Na^+^ insertion/extraction process. In addition, in Figure S5, a plot of current (log (*i*)) versus scan rate (log (*v*)) is obtained by the power–law of *i* = *a × v^b^* (*a* and *b* are adjustable parameters). The Na^+^ storage mechanism can be provided by parameter *b*, dividing surface-controlled redox reaction (when *b* value is about 1) and diffusion-controlled redox reaction (when *b* value is about 0.5) [34,35]. The slopes for NVP@C and NVP bulk are 0.46 and 0.45, respectively, indicating that the NVP crystal has fast sodium ion migration characteristics and the structure of 3D holey-carbon frameworks mainly provides the electron transport. Furthermore, we carried out electrochemical impedance spectroscopy (EIS) analysis to assess the increased electronic and ionic conductivity of NVP@C (Figure 4c). The semicircle in the high frequency region of the Nyquist plots corresponds to the charge transfer resistance (*R*_ct_) at the electrode–electrolyte interface [36]. The *R*_ct_ of NVP@C is much lower than that of NVP bulk, indicating that the charge transfer at the electrode/electrolyte interphase can be significantly facilitated by the intimate holey-carbon framework structure. The interface activity is further analyzed by using a floating test (chronoamperometry) by holding the NVP@C and NVP bulk electrodes at a constant charge voltage of 4.1 V for 1000 s, monitoring the leakage current induced by the interfacial reactions between electrolyte and cathode (Figure 4d). Electrode polarization has an important effect on rapid charge and discharge. The shorter the depolarization time, the faster the electron/ion migration of the electrode, and the better the performance. The leakage current can reflect the interface activity and then represent the time of electrode depolarization, associating with the side reactions between charged cathode and organic electrolyte [37,38]. The leakage current of NVP@C is smaller than that of NVP bulk, indicating that the 3D holey-carbon frameworks possess a stable interface. This significantly reduces the chemical reactivity between NVP@C and the organic electrolyte and bodes well for stable cycle performance and high–rate performance of the NVP@C device.

To sum up the above results, the remarkable electrochemical performance of NVP@C can be attributed to a particular architectural characteristic (Figure 4e). Firstly, magnified details obtained from SEM and TEM images indicate that the NVP@C is aggregated randomly by the NVP nanocrystals, which shorten the ionic diffusion distance when Na^+^ ions transport in the crystal. The charge storage mechanism of Na^+^ does not change with the decrease of NVP particle size, and still maintains a diffusion–controlled redox reaction. Furthermore, the porous structure has a 3D holey-carbon interconnected channel, which can offer a large electrode–electrolyte contact area and high-speed ion transport pathways, greatly facilitating fast Na^+^ transport. The electrode compaction density is favourable and volumetric energy density is superior. Finally, holey-carbon frameworks through the whole electrode guarantee excellent electronic conductivity. Raman and XPS spectra show the existence of a considerable amount of carbon-coating layer, thus favoring electron transport, low charge transfer resistance, small polarization and a stable interface. Therefore, the construction of the 3D holey-carbon frameworks combined with NVP nanocrystals delivers much better electrochemical rate performance and more stable reversible capacity than NVP bulk.

## 4. Conclusions

In summary, a 3D highly interconnected porous Na_3_V_2_(PO_4_)_3_/holey-carbon framework has been successfully synthesized by in-situ polycondensation melamine. A single Na_3_V_2_(PO_4_)_3_ nanocrystal can provide short Na^+^ diffusion distance and holey-carbon frameworks can offer a large electrode–electrolyte contact area and improve electron conductivity. The cathode presents a large capacity of 75.3 mAh g^−1^ at 6000 mA g^−1^ (80 C), improved volumetric energy density (54 Wh L^−1^ for NVP@C) than NVP bulk (31 Wh L^−1^, 6000 mA g^−1^) and a stable capacity of 65.4 mAh g^−1^ after 1000 cycles at 2000 mA g^−1^. Even at low temperature of −15 °C, the NVP@C exhibits a capacity of 60.2 mAh g^−1^ at 1000 mA g^−1^. We envisage that the construct of holey-carbon frameworks combined with single crystal materials can inspire new ideas aimed at high–performance energy storage materials.

## Data Availability

Data is contained within the article or Supplementary Material.

## Supplementary Materials

The following supporting information can be downloaded at: https://www.mdpi.com/article/10.3390/nano13030446/s1, Figure S1: SEM images of (a) NVP bulk and (b) NVP@C; Figure S2: TEM images and SAED pattern of NVP@C; Figure S3: XPS patterns of NVP@C and NVP bulk; Figure S4: Charge–discharge profiles of NVP bulk at different temperatures; Figure S5: Liner fits of peak currents of CV curves with scan rates for NVP@C and NVP bulk.
