# Supplementary material for: One–Step Synthesis of Three–Dimensional Na3V2(PO4)3/Carbon Frameworks as Promising Sodium–Ion Battery Cathode
Source: Nanomaterials (Basel). 2023 Jan 21;13(3):446. doi: 10.3390/nano13030446 (PMC9920691; doi:10.3390/nano13030446)
Supplement: Supplementary file 1 [file nanomaterials-13-00446-s001.zip › nanomaterials-2175677-supplementary.pdf]

Supplementary material

One–Step Synthesis of Three–Dimensional $Na_3V_2(PO_4)_3$/Carbon Frameworks as Promising Sodium–Ion Battery Cathode

Lijiang Zhao [1,2], Xinghua Liu [1], Jinsong Li [1], Xungang Diao [2] and Junying Zhang [1,*]

1 School of Physics, Beihang University, Beijing 100191, China
2 School of Energy and Power Engineering, Beihang University, Beijing 100191, China; diaoxg@buaa.edu.cn
* Correspondence: zjy@buaa.edu.cn

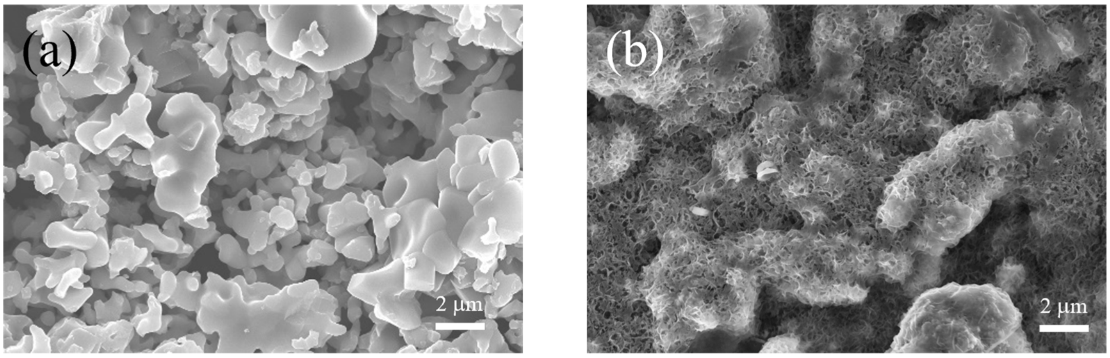

Figure S1. SEM images of (a) NVP bulk and (b) NVP @C.

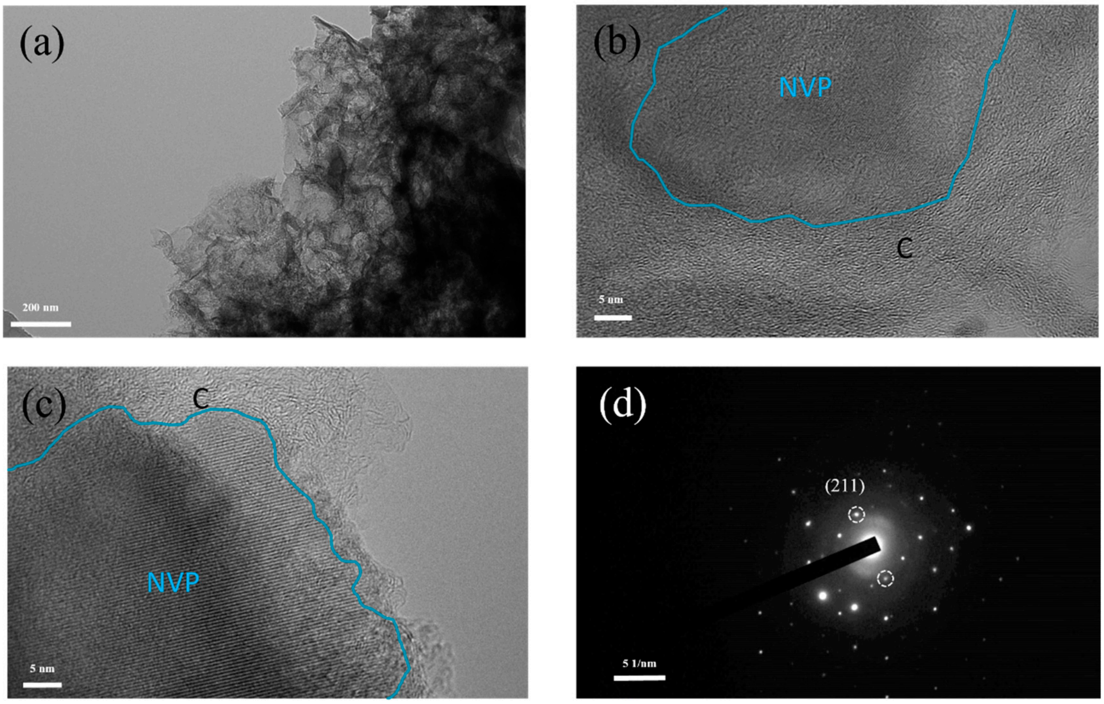

Figure S2. TEM images and SAED pattern of NVP@C.

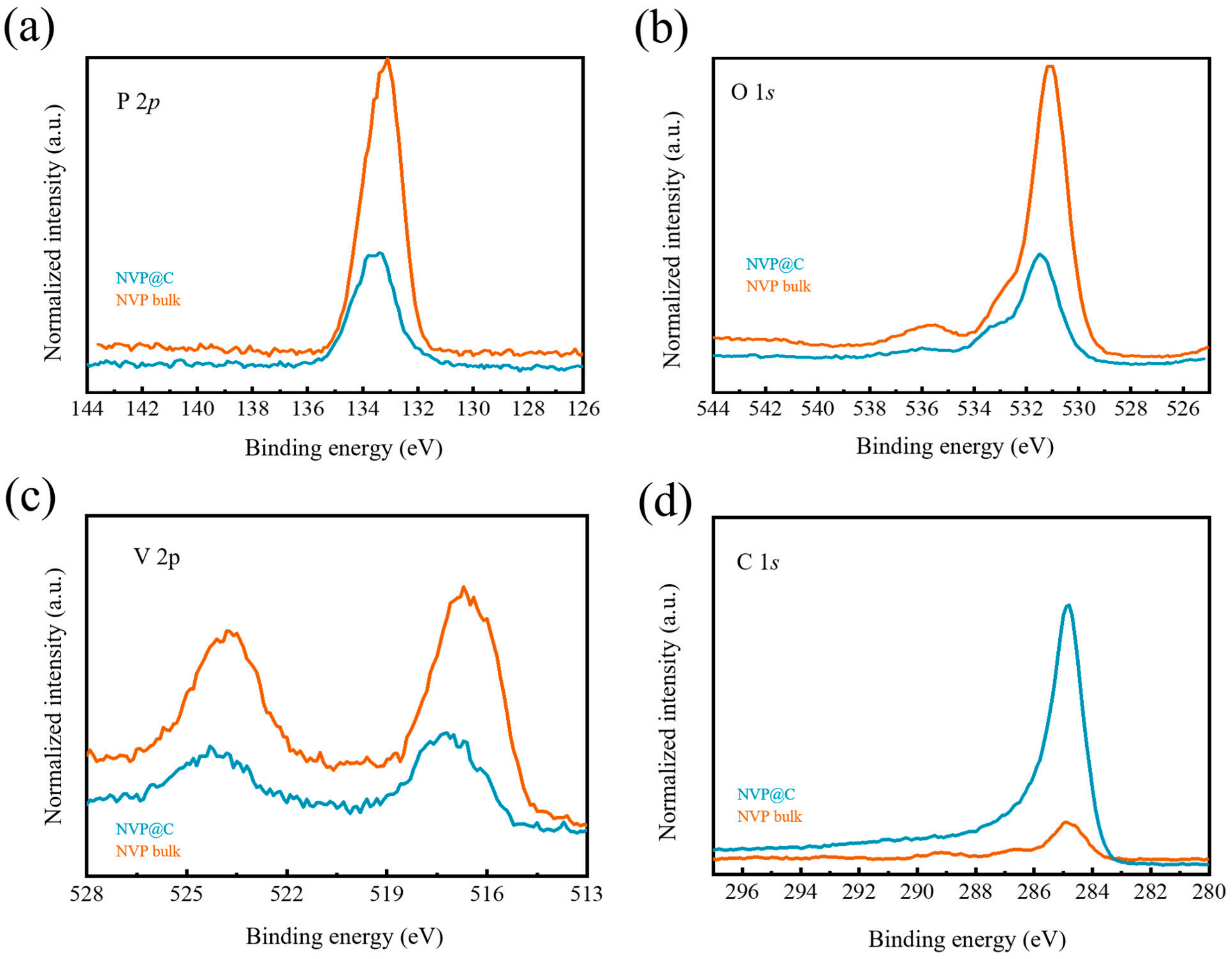

Figure S3. XPS patterns of NVP@C and NVP bulk.

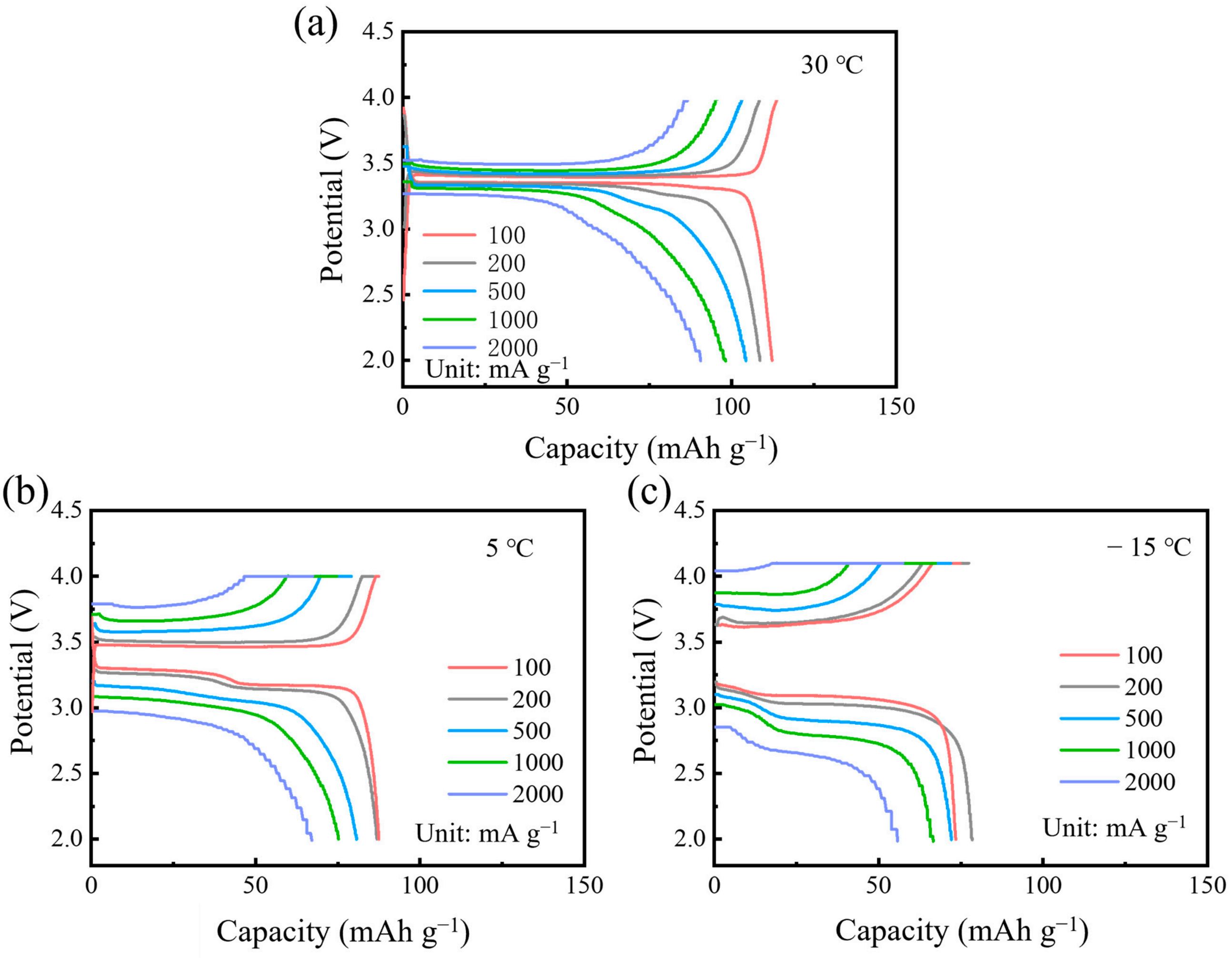

Figure S4. Charge–discharge profiles of NVP bulk at different temperatures.

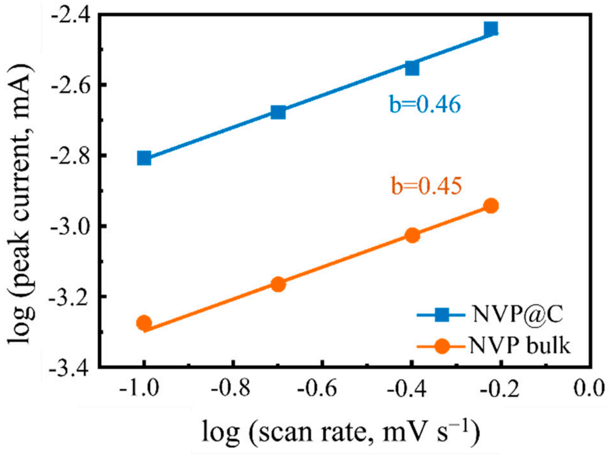

Figure S5. Liner fits of peak currents of CV curves with scan rates for NVP@C and NVP bulk.
